# Association between axial length and choroidal thickness in early age-related macular degeneration

**DOI:** 10.1371/journal.pone.0240357

**Published:** 2020-10-09

**Authors:** Maho Sato, Sakiko Minami, Norihiro Nagai, Misa Suzuki, Toshihide Kurihara, Ari Shinojima, Hideki Sonobe, Kunihiko Akino, Norimitsu Ban, Kazuhiro Watanabe, Atsuro Uchida, Hajime Shinoda, Kazuo Tsubota, Yoko Ozawa

**Affiliations:** 1 Department of Ophthalmology, Keio University School of Medicine, Tokyo, Japan; 2 Laboratory of Retinal Cell Biology, Keio University School of Medicine, Tokyo, Japan; 3 Department of Ophthalmology, St. Luke’s International Hospital, Tokyo, Japan; 4 St. Luke’s International University, Tokyo, Japan; University Hospitals Cleveland, UNITED STATES

## Abstract

The clinical course of age-related macular degeneration (AMD) is related to choroidal conditions, and can be determined by the evaluation of the central choroidal thickness (CCT). The aim of this study was to determine the association between the axial length (AL) and choroidal thickness in AMD by measuring these parameters in patients with and without AMD. Seventy eyes of 70 patients (34 men and 36 women; age, 64–88 years; mean age, 77.0 ± 6.5 years) who underwent cataract surgery from February 2015 to March 2020 at the Department of Ophthalmology, Keio University School of Medicine were retrospectively analyzed. The AMD group (29 patients, 29 eyes) included eyes with early AMD, whereas the control group (41 patients, 41 eyes) included those without ocular diseases other than cataract. Optical coherence tomography images were used to measure the CCT and the choroidal vessel diameter (CVD). The IOL Master was used to measure the AL. The results revealed that mean CCT was greater in the AMD group (238.3 ± 108.3 μm) compared with the age-matched control group (187.2 ± 66.8 μm) (p = 0.03). The CCT was negatively correlated with AL in the overall sample (r = -0.42, p = 0.001), the AMD group (r = -0.42, p = 0.02), and the control group (r = -0.42, p = 0.006). Note that all eyes with CCT > 350 μm were included in the AMD group. CCT and CVD were positively correlated in the overall sample (r = 0.76, p < 0.001) as well as in the individual groups (AMD: r = 0.82, p < 0.001; control: r = 0.76, p = 0.004). Given that CCT is an important parameter for predicting the prognosis of subfoveal diseases, routine evaluation of AL may be valuable for a better understanding of the pathogenesis of AMD.

## Introduction

Due to recent progress in medical science, anti-vascular endothelial growth factor (anti-VEGF) therapy has been developed for the treatment of age-related macular degeneration (AMD). However, AMD is still one of the major causes of blindness in the elderly people, worldwide [1]. In AMD, the lesion spreads from the choroid to the retina, and the choroidal condition may affect the prognosis [2]. Therefore, choroidal research has been attracting attention in the field [2–6]. However, determinants of choroidal condition (such as the thickness), in elderly patients who may be affected with AMD in particular, are not fully understood.

The choroid is a highly vascularized tissue located between the retinal pigment epithelium and sclera. The choroidal vascular system is divided into three layers: choriocapillaris, Sattler’s layer, and Haller’s layer. While the retinal vessels are mainly regulated by the oxygen levels in the body, choroidal vessel dilation are largely related to the neurogenic balance between sympathetic and parasympathetic tensions [7, 8]; this mechanism could be related to the daily variations in the central choroidal thickness (CCT) that is approximately 12 μm [9].

On the other hand, due to the differences in the clinical features, according to the choroidal thickness, among patients who are diagnosed with AMD, the concept of pachychoroid disease spectrum has currently become a topic of interest [4–6]. The term pachychoroid is used to describe abnormal choroidal thickening and/or dilation of the choroidal vessels, and is assumed to be related to choroidal congestion and hyperpermeability. The pachychoroid disease spectrum includes central serous chorioretinopathy, pachychoroid pigment epitheliopathy, pachychoroid neovasculopathy, and polypoidal choroidal vasculopathy (PCV) [6]. These diseases generally occur in elderly patients [2, 10–13]. We have previously reported that the prognosis of one subtype of AMD, PCV, varies according to the pretreatment CCT [2]. Moreover, while CCT is significantly reduced by anti-VEGF therapy, the CCT after the remission of exudative changes from the choroidal lesion ranges approximately from 120 to 220 μm, and differs according to individual patients [2]. Thus, more relevant factors other than the neurogenic factors or VEGF-related mechanism are probably involved in determining CCT in the absence of disease activity.

A rare disease with severe thickening of the choroid, uveal effusion, generally occurs in the eyes with super short axial length (AL) [14]. In contrast, the eyes with high myopia with longer AL have thinner CCT [15]. In addition, the CCT is reported to change and decrease with age [16]. Taken together, the information regarding the relationship between CCT and AL among the elderly people would be of value in understanding AMD pathogenesis.

The aim of the present study was to determine the association between AL and CCT in elderly patients with or without early AMD which had no disease activity. The findings will help understand the determinants of CCT in elderly people and how it is related to the pathogenesis of AMD.

## Materials and methods

### Patients

In this retrospective study, we reviewed the preoperative ophthalmological data for 70 eyes (70 patients; 34 men, 36 women) subjected to cataract surgery between February 2015 and March 2020 at the Medical Retina Division Clinic, Department of Ophthalmology, Keio University Hospital. The study protocol adhered to the tenets of the Declaration of Helsinki and was approved by the Keio University School of Medicine Ethics Committee (Approval number 20100002). In this approval, the ethics committee waived the requirement for written informed consent.

### Inclusion and exclusion criteria

Patients with early AMD (29 patients, 29 eyes) were included in an AMD group, whereas those without ocular diseases other than cataract were included in a control group (41 patients, 41 eyes). The exclusion criteria were as follows: history of exudative changes due to AMD, glaucoma, epiretinal membrane, panretinal photocoagulation, and high myopia (AL ≥ 26.5 mm).

### Eye examinations

All patients underwent complete preoperative ocular examinations, including measurement of the best-corrected visual acuity using a Landolt C chart, fundus biomicroscopy, fundus photography, and spectral domain optical coherence tomography (OCT; Heidelberg Spectralis OCT, Dossenheim, Germany). The central retinal thickness (CRT) was defined as the distance between the inner retinal surface and the inner border of the retinal pigment epithelium, and it was measured using the built-in scale of the OCT system. CCT was defined as the distance between the retinal pigment epithelium and the choroid–sclera boundary, and it was measured using the Enhanced Depth Imaging mode of the OCT system. The vertical diameter of the thickest outer choroidal vessel in Haller’s layer that was within 1000 μm from the fovea was defined as the choroidal vessel diameter (CVD). All measurements were recorded by referring to the scale bars in the OCT system.

### Statistical analyses

All data are expressed as mean ± standard deviation. Pearson’s correlation analysis and the Mann–Whitney U-test were performed using IBM SPSS Statistics for Windows, version 25.0 (IBM Corp., Armonk, N.Y., USA). A p-value of <0.05 was considered statistically significant.

## Results

The patients were aged from 64 to 88 years, with a mean age of 77.0 ± 6.5 years. The mean AL and CCT values were 23.8 ± 1.1 mm and 208.4 ± 90.1 μm, respectively (Table 1). Of these patients, 29 eyes of 29 patients had early AMD and were included in the AMD group (mean age, 78.4 ± 6.8 years), while 41 eyes of 41 patients had cataract only and were included in the control group (mean age, 75.9 ± 6.2 years; Table 1). There were no differences in the age and gender between the groups.

**Table 1:** Characteristics of the patients.

	Total	AMD	Control	p value
		n = 29	n = 41	
Age (range)	77.0 ± 6.5 (64–88)	78.4 ± 6.8 (64–88)	75.9 ± 6.2 (67–88)	0.13
Gender (male [%])	34 [0.54]	16 [0.55]	18 [0.44]	0.34
Axial length (mm)	23.8 ± 1.1	23.7 ± 0.9	23.9 ± 1.2	0.45
CRT (μm)	223.1 ± 36.6	221.2 ± 49.5	224.5 ± 23.4	0.75
CCT (μm)	208.4 ± 90.1	238.3 ± 108.3	187.2 ± 66.8	0.03*
CVD (μm)	107.9±57.6	124.1±71.8	96.5±41.3	0.07

Data are shown in mean ± standard deviation. Mann–Whitney U-test. AMD, age-related macular degeneration; CRT, central retinal thickness; CCT, central choroidal thickness; CVD, choroidal vessel diameter.

*p<0.05.

The mean CCT was significantly greater in the AMD group (238.3 ± 108.3 μm) compared with the control group (187.2 ± 66.8 μm) (p = 0.03, Table 1), and mean CVD had a similar trend (AMD vs control, 124.1 ± 71.8 μm vs 96.5 ± 41.3 μm; p = 0.07, Table 1). There were no differences in the mean AL (p = 0.45, Table 1) and CRT (p = 0.75, Table 1).

**Fig 1:**
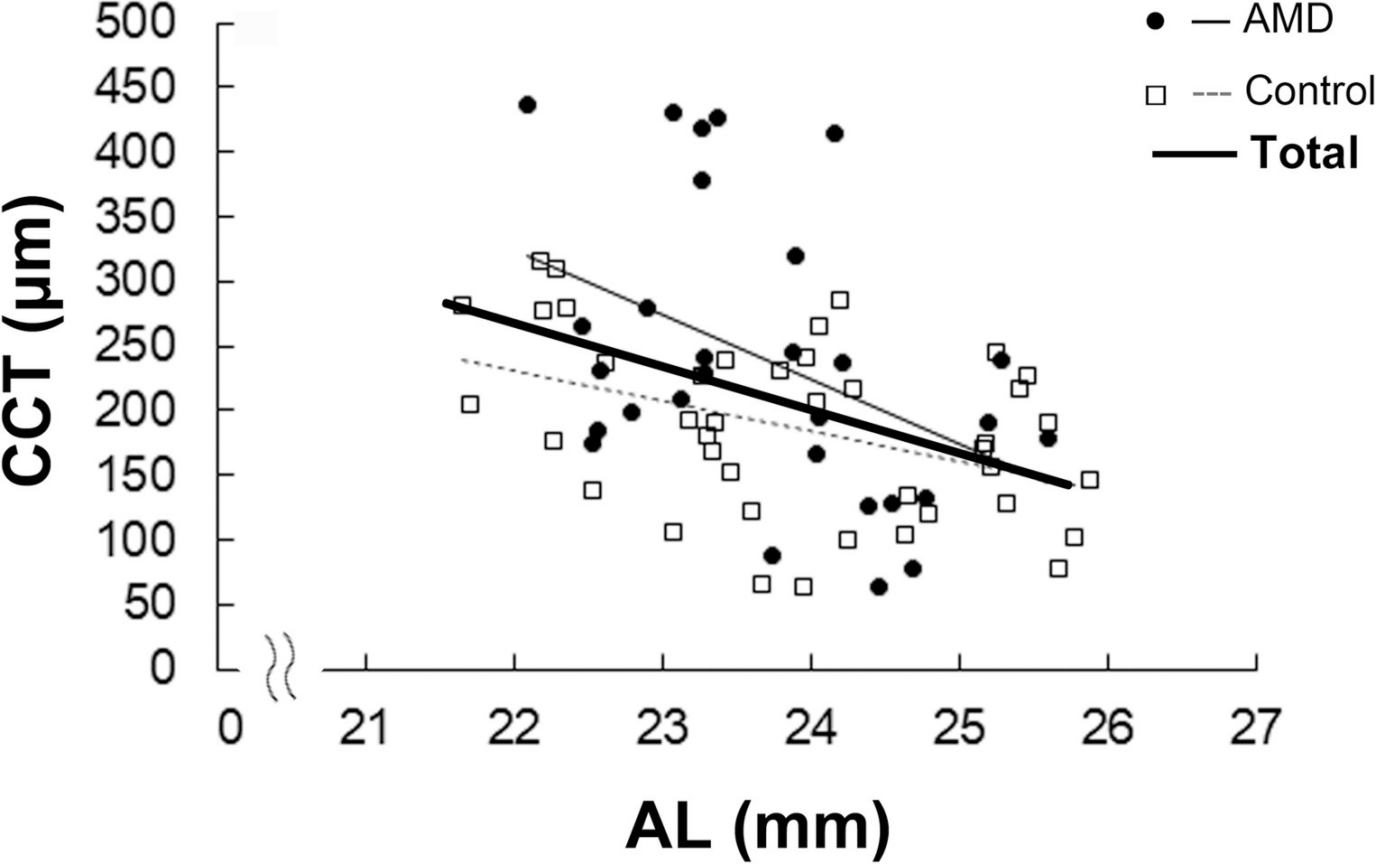
Correlations between CCT and AL. Pearson’s correlation analysis. CCT was negatively correlated with AL in the overall sample as well as in the AMD and control groups. CCT, central choroidal thickness; AL, axial length; AMD, age-related macular degeneration. p < 0.05.

Next, we evaluated the correlations between CCT and AL. In the overall sample, CCT was negatively correlated with AL (r = -0.40, p = 0.001; Fig 1). Negative correlations between CCT and AL were also observed in the AMD group (r = -0.42, p = 0.02; Fig 1) and the control group (r = -0.42, p = 0.006; Fig 1). Note that all the eyes with CCT >350 μm were included in the AMD group (Fig 1).

**Fig 2:**
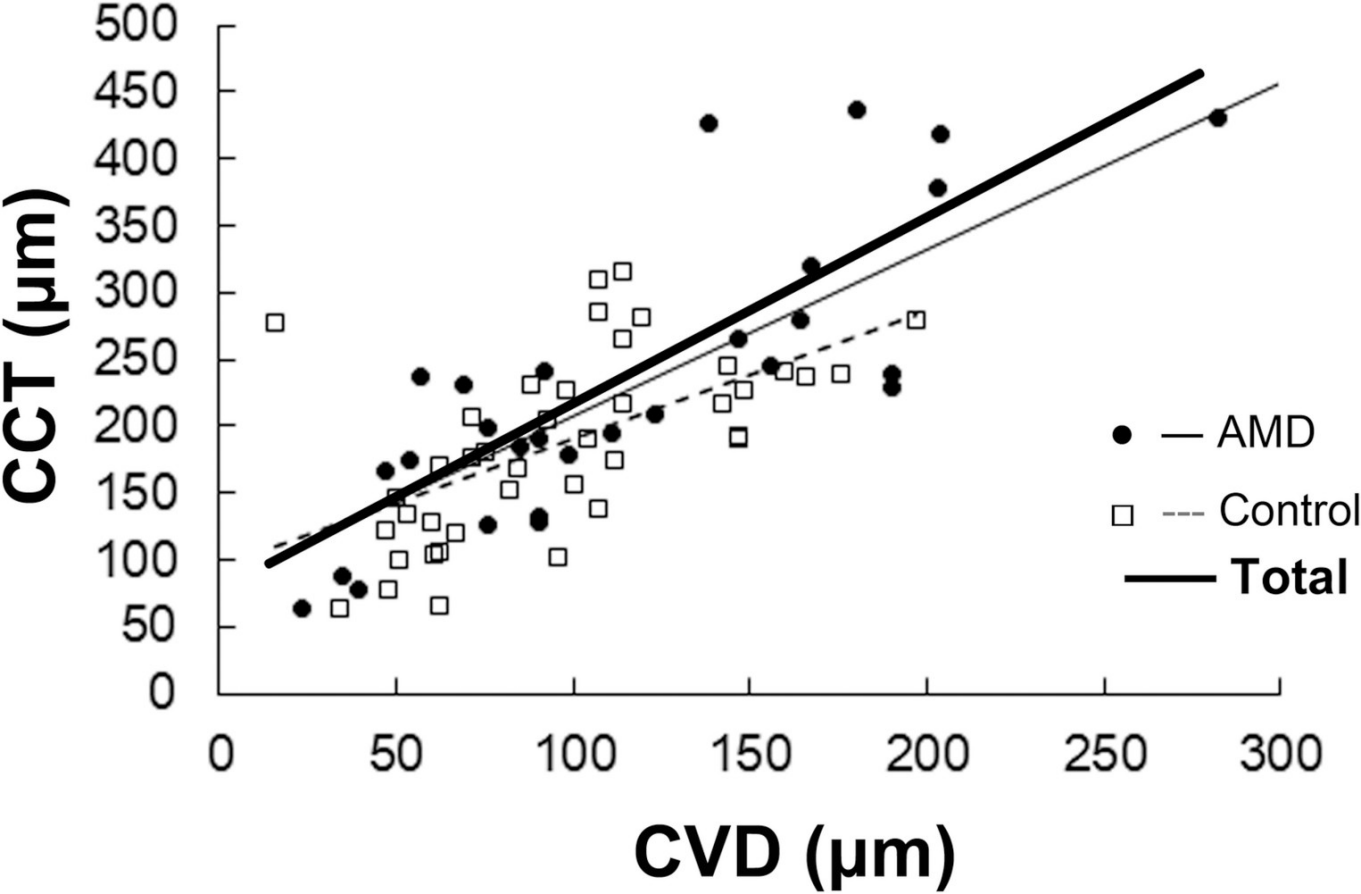
Correlation between CCT and CVD. Pearson’s correlation analysis. CCT was positively correlated with CVD in the overall sample as well as AMD and control groups. CCT, central choroidal thickness; CVD, choroidal vessel diameter; AMD, age-related macular degeneration. p < 0.05.

Also, CCT and CVD were positively correlated in the overall sample (r = 0.82, p < 0.001) as well as the AMD (r = 0.59, p < 0.001) and control (r = 0.76, p = 0.004) groups (Fig 2).

Mean AL was longer in men (24.1 ± 0.97 mm) compared with women (23.5 ± 1.1 mm, p = 0.03; S1 Table), while mean CCT tended to be thinner in men (187.2 ± 87.5 μm) compared with women (228.4 ± 87.8 μm) (p = 0.06; S1 Table). The correlation between CCT and AL was also observed both among men (r = -0.36, p = 0.04) and women (r = -0.37, p = 0.03) (S1 Fig).

There was no correlation between CCT and age in overall sample (p = 0.63; S2 Fig), or either group (AMD group, p = 0.98; control group, p = 0.80; S2 Fig).

## Discussion

In the present study, mean CCT was significantly greater in the AMD group compared with the control group. CCT was negatively correlated with AL in overall group; this finding was also observed when patients with AMD and control were separately analyzed. The eyes with CCT >350 μm were all in the AMD group. Finally, CCT and CVD were positively correlated in patients with and without AMD.

The negative correlation between AL and CCT noted in the present study with elderly people was consistent with the results of previous reports on healthy and relatively young adults (mean age, around 40 years) [17, 18]. Ikuno et al. analyzed healthy adults with a mean age of 39.4 years (range, 23–88 years) while Chen et al. analyzed adults with a mean age of 38 years (range, 30–49 years). In contrast, the present study included patients with a mean age of 77.0 years (range, 64–88 years). The mean CCT values in the studies by Ikuno et al. and Chen et al. were 354 and 334 μm, respectively, whereas that of the present study was 208.4 μm. This is consistent with a previous report of an annual decrease of 3 μm in CCT [16]. An age-related decrease in CCT is mainly observed in the choriocapillaris and Sattler’s layer, with changes also observed in the choroidal large vessel layer (Haller’s layer) [16]. Furthermore, both choroidal vessel and stromal volumes decrease with age [19]. The overall mean CCT in the current study was smaller most likely due to the age-related thinning of the choroid, interestingly, however, there still were correlation between AL and CCT. Moreover, pathological eyes with early AMD also had the negative correlation between AL and CCT.

It is known that pathological choroidal thickening and uveal effusion are caused by the compression of the vortex veins by a thick sclera at the point where the veins outflow through the sclera, and often related to a very short AL [14]. Histological analysis shows that the eyes with very short ALs have abnormal sclera characterized by the disorganization of the collagen fiber bundles and deposits of proteoglycans in the matrix [14]. One of the pachychoroid diseases, central serous chorioretinopathy, may be related to increased flow resistance in the vortex veins that pass through the sclera, resulting in choroidal vessel dilation as shown by angiographies [20]. On the basis of these previous findings and the results of the present study showing that CVD and CCT are positively correlated, CCT increase may be because of choroidal vessel dilation, which could be caused by the increased resistance of outflow through the thick sclera of eyes with short ALs in adults.

Alternatively, previous animal experiments have shown that the changes in AL was associated with light exposure [9], and the decrease in CCT precedes scleral growth during the development of myopia [9, 21]. This suggests that light-induced CCT changes may affect scleral growth, and then, AL. However, further studies are required to validate this suggestion, considering the lack of pathohistological evidence collated over time [22]. Moreover, gene-environment interactions may also play a role in AL elongation and myopia development [23].

Nonetheless, mean CCT was greater and the eyes with CCT>350 μm were all included in the AMD group. Considering that AMD-related SNPs in age-related maculopathy susceptibility 2 [24] and complement factor H [24, 25] are related to choroidal vascular hyperpermeability, and observed in eyes with relatively shorter ALs [24], AMD-related SNPs and AL determinant factors could have the possibility of genetic linkages, although further studies are required.

The limitations of the present study are the relatively small sample size, and the absence of histological data. The fact that early AMD could progress into various subtypes of AMD, such as tAMD and PCV, in the future, could be also a limitation; however, the genetic backgrounds of tAMD and PCV are similar [26–29], and there is a wide overlap in CCT values for individuals with tAMD and PCV, while the mean CCT value is significantly greater for patients with PCV; Koizumi et al. reported that CCT values ranged from 124 to 359 μm in patients with tAMD and from 128 to 404 μm in those with PCV [3]. Future studies with large cohorts are warranted to validate the results of the present study.

In summary, the results of the present study suggest that CCT was negatively correlated with AL and positively correlated with CVD in the elder people with or without early AMD. Given that CCT is an important parameter for predicting the prognoses of subfoveal diseases such as PCV [2], AL data would help in evaluating the outcomes of such diseases. We recommend that AL be routinely measured at the first visit in order to accumulate data for exploring the pathogenesis and disease prognosis in any applicable case.

## Supporting information

S1 Table Characteristics of the male and female patients. Data are shown as mean ± standard deviation. Mann–Whitney U-test. CRT, central retinal thickness; CCT, central choroidal thickness; CVD, choroidal vessel diameter. *p<0.05.
(DOCX)

S1 Fig Correlation between central choroidal thickness (CCT) and axial length (AL). Pearson’s correlation analysis. There were negative correlations between CCT and AL both among men and women. CCT, central choroidal thickness; AL, axial length; AMD, age-related macular degeneration. Control group: patients with no ocular diseases other than cataract.
(TIF)

S2 Fig Correlation between choroidal thickness (CCT) and age. Pearson’s correlation analysis. There is no correlation between CCT and age in the overall sample as well as individual groups (AMD and control groups). CCT, central choroidal thickness; AMD, age-related macular degeneration. Control group: patients with no ocular diseases other than cataract.
(TIF)

## References

[1] Morizane Y, Morimoto N, Fujiwara A, et al Incidence and causes of visual impairment in Japan: the first nation-wide complete enumeration survey of newly certified visually impaired individuals. Jpn J Ophthalmol. 2019 Available: 10.1007/s10384-018-0623-4 30255397

[2] Nagai N, Suzuki M, Minami S, Kurihara T, Kamoshita M, Sonobe H, et al Dynamic changes in choroidal conditions during anti-vascular endothelial growth factor therapy in polypoidal choroidal vasculopathy. Sci Rep. 2019;9: 1–9. 10.1038/s41598-018-37186-2 31388029 PMC6684594

[3] Koizumi H, Yamagishi T, Yamazaki T, Kawasaki R, Kinoshita S. Subfoveal choroidal thickness in typical age-related macular degeneration and polypoidal choroidal vasculopathy. Graefe’s Arch Clin Exp Ophthalmol. 2011;249: 1123–1128. 10.1007/s00417-011-1620-1 21274555

[4] Cheung CMG, Lai TYY, Ruamviboonsuk P, Chen SJ, Chen Y, Freund KB, et al Polypoidal Choroidal Vasculopathy: Definition, Pathogenesis, Diagnosis, and Management. Ophthalmology. 2018;125: 708–724. 10.1016/j.ophtha.2017.11.019 29331556

[5] Stepanov A, Studnička J, Středová M, Jirásková N. Pachychoroid disease of the macula. Ces a Slov Oftalmol. 2018;74: 3–8. 10.31348/2018/1/1-1-2018 30541290

[6] Cheung C.M.G., Lee W.K., Koizumi H, et al Pachychoroid disease. Eye. 10.1038/s41433-018-0158-4 PMC6328576 29995841

[7] Kur J, Newman EA, Chan-Ling T. Cellular and physiological mechanisms underlying blood flow regulation in the retina and choroid in health and disease. Prog Retin Eye Res. 2012;31: 377–406. 10.1016/j.preteyeres.2012.04.004 22580107 PMC3418965

[8] Nickla DL and Wallman J. The multifunctinal choroid. Bone. 2010;23: 1–7. 10.1038/jid.2014.371 25178106 PMC4289436

[9] Ulaganathan S, Read SA, Collins MJ, Vincent SJ. Daily axial length and choroidal thickness variations in young adults: Associations with light exposure and longitudinal axial length and choroid changes. Exp Eye Res. 2019;189: 107850 10.1016/j.exer.2019.107850 31639338

[10] Suzuki M, Nagai N, Izumi-Nagai K, Shinoda H, Koto T, Uchida A, et al Predictive factors for non-response to intravitreal ranibizumab treatment in age-related macular degeneration. Br J Ophthalmol. 2014;98: 1186–1191. 10.1136/bjophthalmol-2013-304670 24711658 PMC4145467

[11] Suzuki M, Nagai N, Shinoda H, Uchida A, Kurihara T, Tomita Y, et al Distinct Responsiveness to Intravitreal Ranibizumab Therapy in Polypoidal Choroidal Vasculopathy with Single or Multiple Polyps. Am J Ophthalmol. 2016;166: 52–59. 10.1016/j.ajo.2016.03.024 27017997

[12] Nagai N, Suzuki M, Uchida A, Kurihara T, Kamoshita M, Minami S, et al Non-responsiveness to intravitreal aflibercept treatment in neovascular age-related macular degeneration: Implications of serous pigment epithelial detachment. Sci Rep. 2016;6: 1–10. 10.1038/s41598-016-0001-8 27403807 PMC4939600

[13] Sasaki M, Kawasaki R, Uchida A, Koto T, Shinoda H, Tsubota K, et al Early signs of exudative age-related macular degeneration in Asians. Optom Vis Sci. 2014;91: 849–853. 10.1097/OPX.0000000000000317 24978864 PMC4186733

[14] Uyama M, Takahashi K, Kozaki J, Tagami N, Takada Y, Ohkuma H, et al Uveal effusion syndrome1: Clinical features, surgical treatment, histologic examination of the sclera, and pathophysiology. Ophthalmology. 2000;107: 441–449. 10.1016/s0161-6420(99)00141-4 10711879

[15] Flores-Moreno I, Lugo F, Duker JS, Ruiz-Moreno JM. The Relationship Between Axial Length and Choroidal Thickness in Eyes With High Myopia. Am J Ophthalmol. 2013;155: 314-319.e1. 10.1016/j.ajo.2012.07.015 23036569

[16] Wakatsuki Y, Shinojima A, Kawamura A, Yuzawa M. Correlation of aging and segmental choroidal thickness measurement using swept source optical coherence tomography in healthy eyes. PLoS One. 2015;10: 1–14. 10.1371/journal.pone.0144156 26632821 PMC4669163

[17] Ikuno Y, Kawaguchi K, Nouchi T, Yasuno Y. Choroidal thickness in healthy Japanese subjects. Investig Ophthalmol Vis Sci. 2010;51: 2173–2176. 10.1167/iovs.09-4383 19892874

[18] Michalewski J, Michalewska Z, Nawrocka Z, Bednarski M, Nawrocki J. Correlation of choroidal thickness and volume measurements with axial length and age using swept source optical coherence tomography and optical low-coherence reflectometry. Biomed Res Int. 2014;2014 10.1155/2014/639160 25013793 PMC4075071

[19] Sonoda S, Sakamoto T, Yamashita T, Uchino E, Kawano H, Yoshihara N, et al Luminal and stromal areas of choroid determined by binarization method of optical coherence tomographic images. Am J Ophthalmol. 2015;159: 1123-1131.e1. 10.1016/j.ajo.2015.03.005 25790737

[20] Kishi S, Matsumoto H, Sonoda S, Hiroe T, Sakamoto T, Akiyama H. Geographic filling delay of the choriocapillaris in the region of dilated asymmetric vortex veins in central serous chorioretinopathy. PLoS One. 2018;13: 1–12. 10.1371/journal.pone.0206646 30412594 PMC6226146

[21] Wallman J, Mcfadden S. Monkey eyes grow into focus. Nat Med. 1995. 10.1038/nm0895-737 7585168

[22] Mansour AM. Unmeasurable small size superficial and deep foveal avascular zone in nanophthalmos: the Collaborative Nanophthalmos OCTA Study. Br J Ophthalmol. 2019. 10.1136/bjophthalmol-2018-312781 30322954

[23] Cooper J, Tkatchenko A V. A Review of Current Concepts of the Etiology and Treatment of Myopia. Eye Contact Lens. 2018;44: 231–247. 10.1097/ICL.0000000000000499 29901472 PMC6023584

[24] Yoneyama S, Sakurada Y, Kikushima W, Sugiyama A, Tanabe N, Mabuchi F, et al Genetic factors associated with choroidal vascular hyperpermeability and subfoveal choroidal thickness in polypoidal choroidal vasculopathy. Retina. 2016;36: 1535–1541. 10.1097/IAE.0000000000000964 26745149

[25] Hosoda Y, Yoshikawa M, Miyake M, Tabara Y, Ahn J, Woo SJ, et al CFH and VIPR2 as susceptibility loci in choroidal thickness and pachychoroid disease central serous chorioretinopathy. Proc Natl Acad Sci U S A. 2018;115: 6261–6266. 10.1073/pnas.1802212115 29844195 PMC6004488

[26] Fan Qiao. Shared genetic variants for polypoidal choroidal vasculopathy and typical neovascular age-related macular degeneration in East Asians. J Hum Genet. 2017. 10.1038/jhg.2017.83 28835638

[27] Chen H, Liu K, Chen LJ, Hou P, Chen W, Pang CP. Genetic associations in polypoidal choroidal vasculopathy: A systematic review and meta-analysis. Mol Vis. 2012;18: 816–829. 22509112 PMC3324368

[28] Handy Diane E., Rita Castro JL. Three Major Loci Involved in Age-Related Macular Degeneration are also Associated with Polypoidal Choroidal Vasculopathy. Bone. 2011;23: 1–7. 10.1161/CIRCULATIONAHA.110.956839 20378180 PMC2901561

[29] Dansingani KK, Gal-Or O, Sadda SR, Yannuzzi LA, Freund KB. Understanding aneurysmal type 1 neovascularization (polypoidal choroidal vasculopathy): a lesson in the taxonomy of ‘expanded spectra’–a review. Clin Exp Ophthalmol. 2018;46: 189–200. 10.1111/ceo.13114 29178419 PMC5900982

