# Supplementary material for: Association between axial length and choroidal thickness in early age-related macular degeneration
Source: PLoS One. 2020 Oct 9;15(10):e0240357. doi: 10.1371/journal.pone.0240357 (PMC7546466; doi:10.1371/journal.pone.0240357)
Supplement: S1 Table — Data are shown as mean ± standard deviation. Mann–Whitney U-test. CRT, central retinal thickness; CCT, central choroidal thickness; CVD, choroidal vessel diameter. *p<0.05. (DOCX) [file pone.0240357.s001.docx]

| **S1 Table Characteristics of the male and female patients** | | | |
| --- | --- | --- | --- |
|  | **Male** | **Female** | **p value** |
|  | n=34 | n=36 |  |
| Age | 77.8 ± 6.5 | 76.2 ± 6.5 | 0.31 |
| Axial length（mm） | 24.1 ± 0.97 | 23.5 ± 1.1 | 0.03* |
| CRT（μm） | 221.8 ± 23.8 | 224.4 ± 45.5 | 0.76 |
| CCT（μm） | 187.2 ± 87.5 | 228.4 ± 87.8 | 0.06 |
| CVD（μm） | 101.7 ± 61.2 | 113.8 ± 53.3 | 0.38 |
| Data are shown as mean ± standard deviation. Mann-Whitney U-test. CRT, central retinal thickness; CCT, central choroidal thickness; CVD, choroidal vessel diameter. *p<0.05. | | | |
