# Supplementary figures and images for: Association between axial length and choroidal thickness in early age-related macular degeneration
Source: PLoS One. 2020 Oct 9;15(10):e0240357. doi: 10.1371/journal.pone.0240357 (PMC7546466; doi:10.1371/journal.pone.0240357)

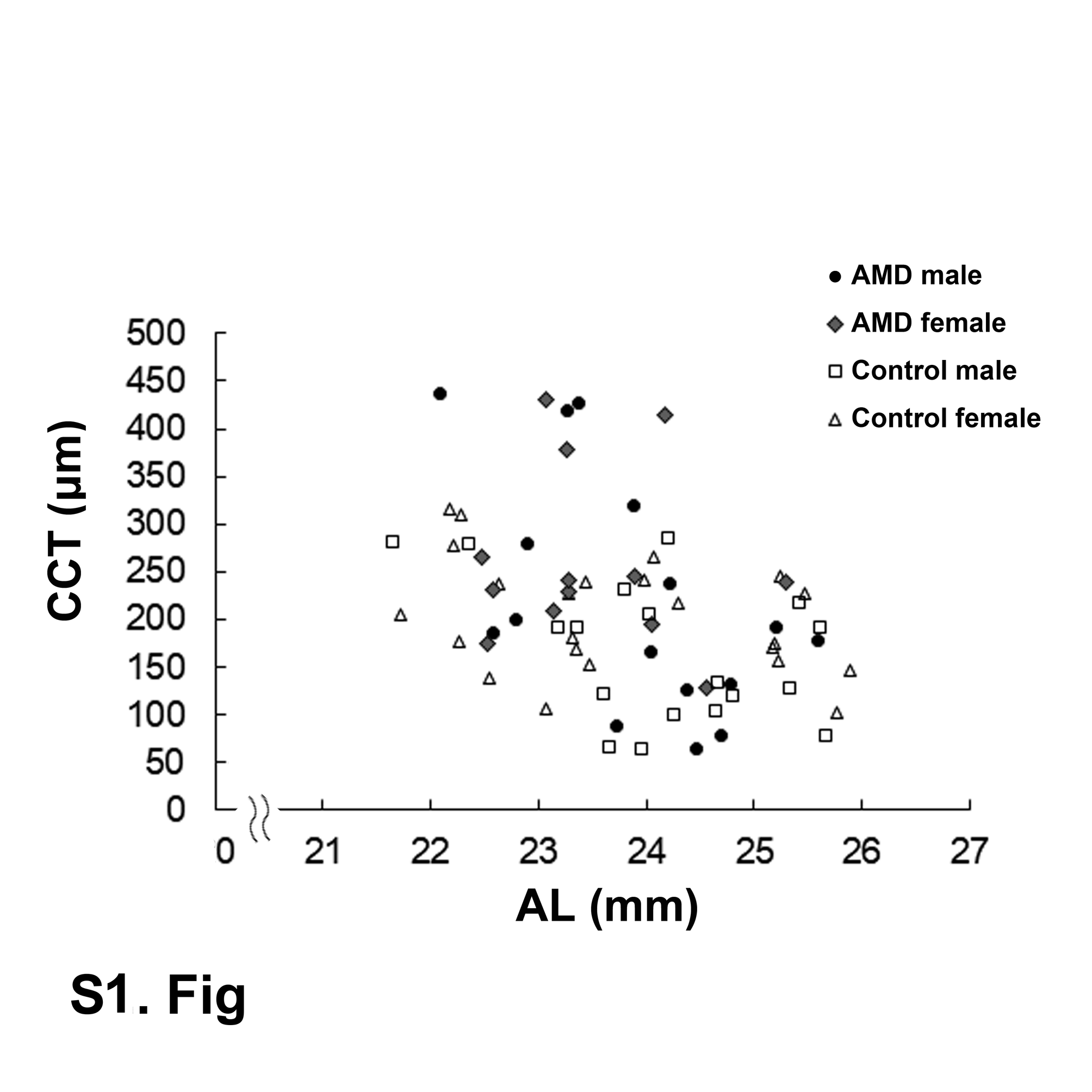

Supplement: S1 Fig — Pearson’s correlation analysis. There were negative correlations between CCT and AL both among men and women. CCT, central choroidal thickness; AL, axial length; AMD, age-related macular degeneration. Control group: patients with no ocular diseases other than cataract. (TIF) [file pone.0240357.s002.tif]

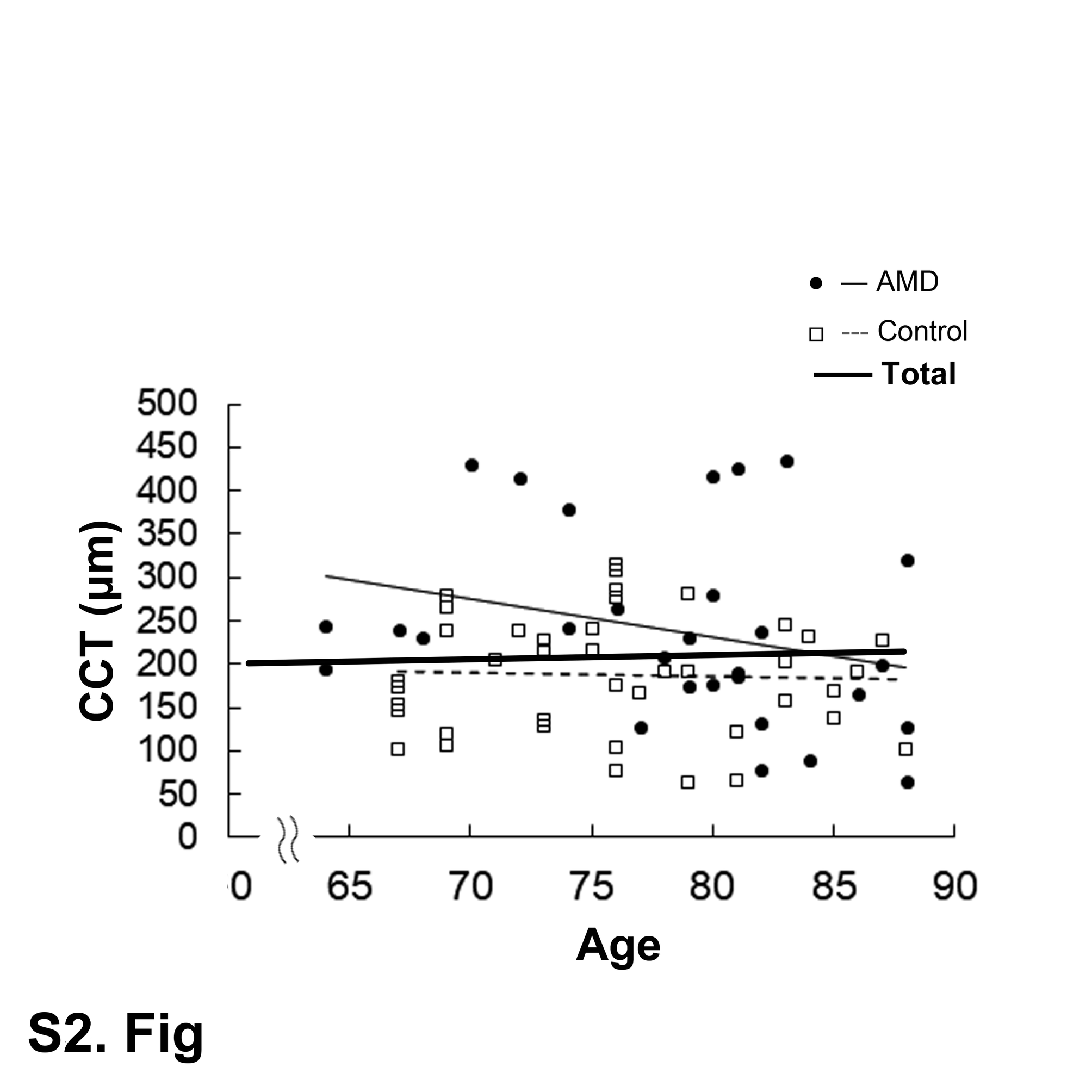

Supplement: S2 Fig — Pearson’s correlation analysis. There is no correlation between CCT and age in the overall sample as well as individual groups (AMD and control groups). CCT, central choroidal thickness; AMD, age-related macular degeneration. Control group: patients with no ocular diseases other than cataract. (TIF) [file pone.0240357.s003.tif]
